# A Wireless Sensor Network-Based Portable Vehicle Detector Evaluation System

**DOI:** 10.3390/s130101160

**Published:** 2013-01-17

**Authors:** Seong-eun Yoo

**Affiliations:** School of Computer and Communication Engineering, Daegu University, Jilyang-up, Gyeongsan, Gyeongbuk 712-714, Korea; E-Mail: seyoo@daegu.ac.kr; Tel.: +82-53-850-6644; Fax: +82-53-850-6629

**Keywords:** traffic monitoring, reference instrument, vehicle detection system, wireless sensor networks, VDS, WSN

## Abstract

In an upcoming smart transportation environment, performance evaluations of existing Vehicle Detection Systems are crucial to maintain their accuracy. The existing evaluation method for Vehicle Detection Systems is based on a wired Vehicle Detection System reference and a video recorder, which must be operated and analyzed by capable traffic experts. However, this conventional evaluation system has many disadvantages. It is inconvenient to deploy, the evaluation takes a long time, and it lacks scalability and objectivity. To improve the evaluation procedure, this paper proposes a Portable Vehicle Detector Evaluation System based on wireless sensor networks. We describe both the architecture and design of a Vehicle Detector Evaluation System and the implementation results, focusing on the wireless sensor networks and methods for traffic information measurement. With the help of wireless sensor networks and automated analysis, our Vehicle Detector Evaluation System can evaluate a Vehicle Detection System conveniently and objectively. The extensive evaluations of our Vehicle Detector Evaluation System show that it can measure the traffic information such as volume counts and speed with over 98% accuracy.

## 1. Introduction

With advancements in semiconductor and embedded software technologies, wireless sensor networks (WSNs) have been applied in diverse industrial fields. Well-known WSN applications include consumer electronics, home or factory automation, personal healthcare, asset management [1], intelligent agriculture [2], and industrial control and monitoring [3]. Another application of WSNs is in intelligent transportation systems (ITS) where WSNs provide useful information such as real-time traffic information and statistics for both drivers and the transportation bureau [4–11]. This paper proposes an application of a WSN evaluating deployed Vehicle Detection Systems (VDSs) for smart and safe transportation systems.

VDSs are one of the basic building blocks of the upcoming smart transportation environment, and maintaining the performance of VDSs is crucial to providing a required level of service. The conventional way to maintain the performance of a VDS is to regularly compare the traffic information from the installed VDS with that gathered by a portable VDS (*i.e.*, a reference instrument), and to adjust the installed VDS based on the evaluation results [12]. The existing portable VDS can be one of several types—a video-frame detector, tape-switch (or piezoelectric), laser detector [13], or loop detector—according to the technology utilized to detect a vehicle [14,15]. The video-frame detector requires an expert to analyze the recorded images, which can be up to 30 frames per second. Video-frame detectors are one of the most credible VDS methods, but without a skilled expert, the method is error-prone [13]. The other types of VDS are wired, and it takes a long time to install them in the pavement. Vehicles can also easily damage them. In addition, the first three types of VDS cannot be used easily to detect multiple lanes of traffic at the same time. This scalability is becoming one of the important requirements for VDS evaluation systems with the increase of multi-lane roads [16]. Recently, wireless VDSs have begun to be used in the field, but they cannot monitor traffic in real-time. Therefore, to attack and eliminate the aforementioned defects of existing VDS evaluation systems, this paper proposes a novel architecture for a Portable Vehicle Detector Evaluation System (PES) based on a WSN considering the requirements of a new portable evaluation system (Table 1). Our PES consists of T-Sensor nodes, a T-Sink node, a T-BS-com node, a WSN, and the T-Mon host. T-Sensor nodes, enclosed in slim and hard cases, are taped to the pavement. Each T-Sensor node equipped with a magneto-resistive sensor [17–19] detects the disturbance of the Earth magnetic field caused by a vehicle and identifies the approach or the departure of the vehicle with a detection algorithm. It sends the event packet to T-Sink node. The T-Sink node relays the packets to the T-BS-com node, and the T-BS-com node bridges the WSN and the T-Mon host, which is the main controller of the PES. We describe the architecture and design of PES in detail. Through extensive experimental evaluation, we demonstrate the PES to be a feasible reference instrument, since it alleviates the problems of the existing evaluation systems and can measure traffic information with over 98% accuracy. In addition, PES shows better performance than a commercial wireless sensor network-based VDS.

We may summarize the contributions of this paper as follows: (1) it proposes a novel application of wireless sensor networks, (2) it presents the architecture of an evaluation system for VDS, and (3) it demonstrates the feasibility of a wireless sensor network-based reference instrument through extensive evaluation of a real-world PES implementation.

The rest of the paper is organized as follows: Section 2 surveys and compares the conventional methods or instruments used to evaluate a VDS. The system architecture and design of PES are presented in Section 3. The results of implementing and experimenting with our PES are analyzed in Section 4. Finally, Section 5 concludes the paper with a summary and discussion about future work.

## 2. Related Work

In this section, we summarize the existing VDS evaluation systems. Although there are stationary VDS evaluation systems, this paper focuses on portable ones, since most VDSs have already been installed at a stationary site, so we cannot move them to where the stationary evaluation system is installed. The existing portable VDS evaluation systems are based on several kinds of detection technologies. The most commonly-used evaluation system utilizes image frame analysis with a camera [13,19]. The video camera is installed at the same place where the VDS to be evaluated is installed. When a vehicle passes a known distance as shown in the video, the travel time is measured in frame units. The number of frames per second is used to measure the distance travelled by the vehicle (1 frame = 1/30 s). However, an expert measures the traverse time as an integer number of frames, and that expert must personally evaluate any fractions of frames. It is difficult for different experts (*i.e.*, the video frame analyzers) to measure the fractions of the number of frames consistently and objectively, which adds variability to the results. In addition, image frame analysis tools sometimes experience mechanical failures.

Another type of portable evaluation system is based on laser detectors [14]. This evaluation system sends laser light to a reflector, which is stuck to the pavement. The signal hits the reflector, which reflects it to the evaluation system receiver. With this transmitter and receiver set, the evaluation system can detect a passing vehicle. To measure the speed of a vehicle, the evaluation system needs two sets of transmitters and receivers separated by a known distance. A laser evaluation system can detect the traverse time of a vehicle very accurately without any room for subjectivity. However, since the evaluation system must be deployed on the side of the road and the reflector attached to the lane with the VDS to be evaluated, it is easy for any passing vehicle to interfere with the laser signal. In other words, only the traffic of the outer lane can be measured with this system and the measurement of multiple lanes (*i.e.*, scalability) is very difficult.

The third type of evaluation system is designed with a tape-switch or piezoelectric cable [13]. Passing vehicles press down onto the piezoelectric cable, which creates an electrical impulse. If two piezoelectric cables are separated by a known distance, traffic information including volume count, speed, and occupancy can be measured. Although this type of evaluation system can overcome the drawbacks of video frame detection and can measure traffic information very accurately, the piezoelectric cable is relatively fragile and can be easily broken. It is also difficult for a wired cable system to measure multiple lanes of traffic.

The last types of evaluation systems can be designed with helps of other sensors such as Passive Infrared (PIR) or magneto-resistive (MR) sensors [20]. A PIR detects the radiation emitted from vehicles and road surfaces, since any object with a temperature higher than absolute zero emits radiation in the far IR part of the electromagnetic spectrum depending on the object's surface temperature, size and structure [20]. However, PIR performance is highly degraded by the environmental factors such as sunlight, fog, rain, and atmosphere, and the overall speed accuracy measured on a PIR sensor is reported as 90% [20], which is quite poor for an evaluation system. Recently, a magneto-resistive senor has been utilized in a vehicle detection system [21,22]. As long as simple self-calibration and detection algorithms are adopted, it can detect the disturbance of the Earth's magnetic field caused by vehicles despite slow changes in the ambient magnetic field caused by environmental factors. Since the MR sensor is small and draws a low average current with the help of duty cycling or other wakeup sensors such as an optical sensor [23], it is adopted in wireless sensor nodes. Some papers report the research results on WSN applications to intelligent transportation systems for safe driving, traffic monitoring and control, but their main concerns are early warning of potential dangerous situations [24], signal control with traffic information gathered by in-vehicle sensor nodes [25], and traffic estimation and control with small sets of sensor nodes [26] rather than evaluation of other VDSs. By now some VDS based wireless sensor networks have been developed, but no evaluation system based on them proposed yet meets all of the requirements (Table 1).

Although there are some pilot programs for traffic measurement based on a wireless sensor node with a magneto-resistive sensor, the accuracy, portability, and the traceability are not satisfactory and the systems without fault-tolerance are prone to malfunction and accuracy degradation due to wireless interference [21,22,27]. Therefore, to attack the aforementioned problems of existing VDS evaluation systems, we propose our PES meeting the requirements (Table 1) of a portable VDS evaluation system. Our PES operates autonomously, so unlike video analysis, the evaluation is objective. In addition, unlike laser or piezoelectric cable evaluation systems, the PES can easily scale up to eight lanes of traffic at a time. Finally, comparing the existing WSN-based VDS, our PES offers high accuracy, portability, traceability, and fault-tolerance. Table 2 compares the existing portable evaluation systems and the proposed PES. For the cost, referring to [20,28], we compare the minimum device costs for some types of evaluation systems assuming two lanes. PES is the most cost-effective, since the device cost of the PES is $200 × 2 × 2 + $1,200 = $2,000, while the costs for a video image processing-based VDS and a laser based one (excluding video recording system) are $4,900 and $31,580, respectively.

## 3. System Architecture and Design

In this section, we describe the system architecture and detailed design of our PES from a top-down approach. Rather than mentioning evaluated vehicle detection systems, this section focuses on the proposed PES itself.

### 3.1. Overall System

**Figure 1.**
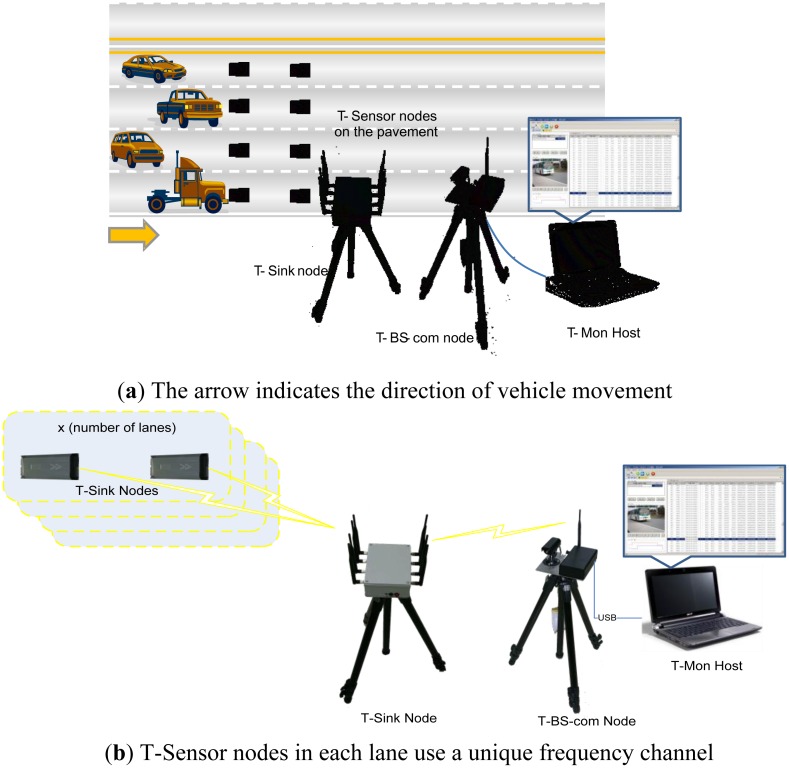
(**a**) Overall system description (**b**) System description with network connections.

As described in Figure 1, the PES consists of a Telematics Sensor Network (TSN) and a T-Mon host PC. Wireless Sensor Network (WSN) is still more general terminology, but we call the network architecture TSN due to the unique characteristics of the proposed application.

A TSN is a network of T-Sensor nodes, a T-Sink node, and a T-BS-com node with a video camera. The PES is deployed in the place where the VDS under evaluation is installed. A pair of T-Sensor nodes is taped to the surface of the road with strong stickers. Whenever each node detects any vehicle event (APPROACH or DEPARTURE), it reports the event to the T-Sink node through the pre-assigned channel. When the T-Sink node receives the event packets, it takes the timestamp for each event packet, appends the timestamp to each event packet, and forwards it to the T-BS-com node. All of the packets including event packets are delivered to the T-Mon Host via the T-BS-com node, and the T-Mon host estimates traffic information such as speed, volume, and occupancy for each lane based on the received event packets. The T-Mon host shows the current communication and battery status and traffic information measured and estimated by PES in real-time and saves the information for comparison with the performance of the evaluated VDS. We present a detailed explanation in the following sub-sections.

### 3.2. Telematics Sensor Network

The most essential component of the PES is the TSN. The TSN is comprised of T-Sensor nodes, T-Sink nodes, and a T-BS-com node. In this sub-section, we describe the network architecture of the PES first, and the design of each kind of node follows.

**Figure 2.**
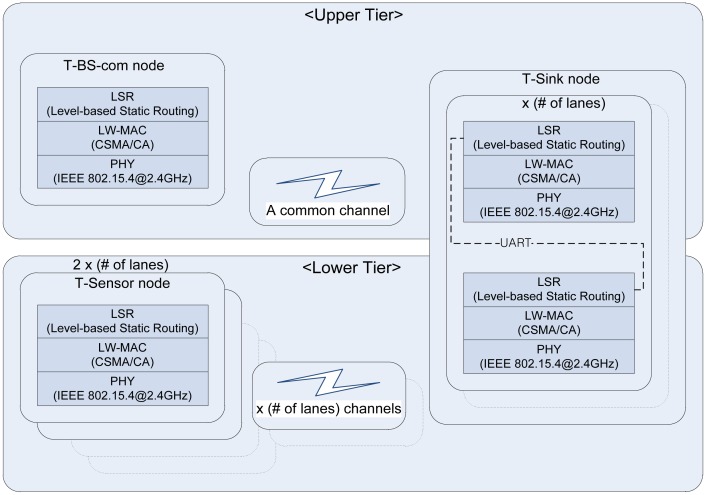
Network architecture of TSN.

The network architecture (Figure 2) of the PES is based on that of S3 [29], a two-tiered architecture targeting simple and stable operation. However, it is enhanced to meet the new requirements of the PES such as 1-hop broadcast packet support, event packet retransmission, and time synchronization. The lower tier network is used for communication between T-Sensor nodes and the T-Sink node. It is time-critical and easily interfered by the vehicles. To avoid packet collision between different lanes, T-Sensor nodes in each lane are assigned a unique frequency channel. The Physical (PHY) layer of TSN is compatible with IEEE 802.15.4-2003 (2.4 GHz). Normal packets are delivered to the T-Sink node through Lightweight MAC (LW-MAC) based on a simple CSMA/CA algorithm similar to the unslotted CSMA/CA of IEEE 802.15.4 [30]. If the MAC-level ACK frame is not received from the receiver for the ACK wait duration, the sender identifies the packet lost and retransmit the lost packet in the MAC-level. However, LW-MAC treats the event packets differently, and the event packets are transmitted to the T-Sink node without Clear Channel Assessment (CCA), random back-off, and MAC-level retransmission. If an event packet from a T-Sensor node fails to be delivered to the T-Sink node (*i.e.*, the MAC-level ACK frame for the packet is not received from the T-Sink node to the T-Sensor node for the ACK wait duration), now the application layer of the T-Sensor node (instead of the MAC layer) tries to retransmit the failed event packet after updating the delay field of the original packet to accommodate the retransmission latency. Another function of LW-MAC is to take the received time stamp and add it to the header of a buffer retaining the received packet. The time stamp is used to measure the traverse time of a vehicle between two consecutive T-Sensor nodes in the same lane. By measuring the traverse time in the T-Sink node, time synchronization between the T-Sensor nodes is not required.

**Figure 3.**
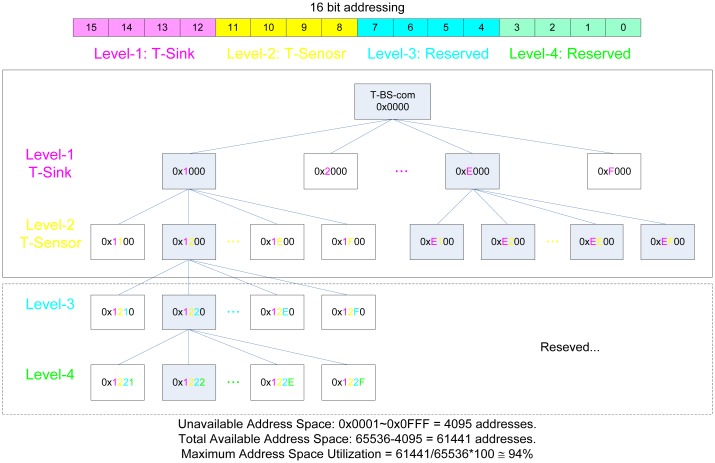
Addressing Scheme for Level-based Static Routing.

All packets are routed to their destination using the Level-based Static Routing (LSR) protocol, which is based on level-based static addressing, for fast and robust routing. The level-based static addressing scheme allocates a 16-bit address to each node so as to assign each level to each nibble of the 16 bit address as described in Figure 3. By sacrificing 6% of the address space, the LSR protocol gains the advantages of simplicity, speed, and table-less routing.

The upper tier network is used for data exchange between the T-Sink nodes and the T-BS-com node. The network uses a common frequency channel different from the channels used by the T-Sensor nodes. Every packet exchanged between them is delivered over IEEE802.15.4-2003 PHY, LW-MAC, and LSR. The packets over the upper tier network are not time-critical, so the LW-MAC in the tier is configured to use normal CSMA/CA and multiple retransmissions.

In the case of T-Mon Host failure, T-BS-com saves every traffic information packet in its local storage. The saved information can be retrieved to evaluate the VDS after the recovery of the T-Mon Host. In addition, in the case of T-Sink node failure, every event generated by a T-Sensor node is saved in its local Secure Digital (SD) card. Any undelivered traffic information of the T-Sensor node can be restored during post-processing of the locally-saved events. To do that, time synchronization between two T-Sensor nodes in the same lane is required. Since the maximum operation time of PES is less than 6 hours and the accuracy of the Temperature Compensated Crystal Oscillator (TCXO) used is 2.5 parts per million (ppm) between −30 °C and 75 °C, with a single time synchronization packet exchange, the worst possible time offset during a 10 minute period between two T-Sensor nodes in the same lane is [10 × 60 × (2.5/(10^6^) = 1.5 ms] + 1.5 ms, or 3 ms. Although periodic handshaking is necessary to maintain the required time synchronization accuracy, a single synchronization scheme is designed and implemented for an operation time of 6 hours for this pilot project.

Before explaining the time synchronization details, we will briefly outline the timer operation in the T-Sensor and T-sink nodes. In PES, the continuous mode of Timer_A is used among various timer operation modes of MSP430 [31]. In the continuous mode, the Timer_A Register (TAR) is repeatedly increased to 0xFFFF and restarts from zero, according to the timer clock. Whenever TAR reaches the value of Timer_A Capture/Compare 0 Register (TACCR0), a TACCR0 interrupt is triggered. In the Interrupt Service Routine (ISR) for the interrupt, a global variable, *u32Tick* (a 32-bit software time tick), is increased by 1 and 0x0A00 is added to TACCR0 to generate the next periodic timer interrupt with a period of 0x0A00 counts on the timer clock. With this configuration, assuming an 8MHz timer clock, TAR increases every 0.125 μs, and 0x0A00 counts generates a 0.125 × 0x0A00 = 320 μs periodic timer interrupt.

**Figure 4.**
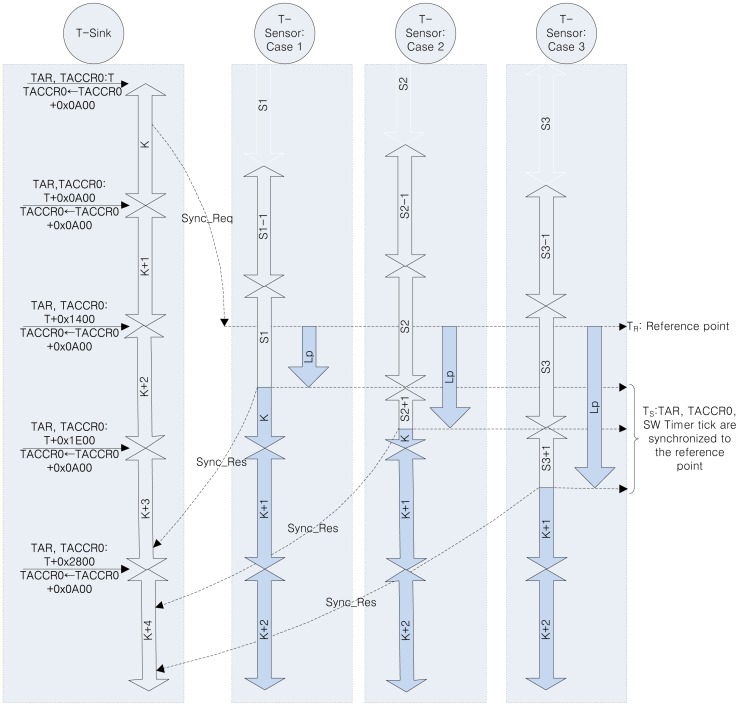
Timing diagram of the synchronization procedure of T-Sensor nodes to the T-sink node.

**Figure 5.**
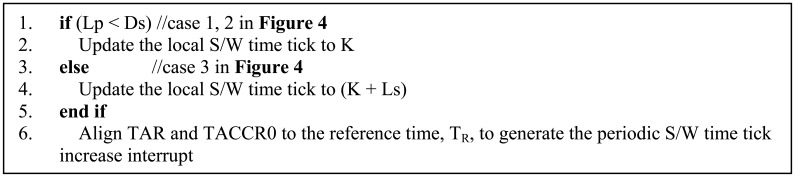
Procedure for T-Sensor node to synchronize its local software time tick to the received Sync_Req broadcast message.

The detailed procedure is explained in Figures 4 and 5, and the notations used in the figures are summarized in Table 3. In PES, data on the second, minute, hour, day, month, and year is distributed from the T-Mon Host to the T-Sensor nodes via T-Sink nodes using so-called sender-receiver synchronization, but the time tick synchronization between T-Sensor nodes in the same lane is achieved by a broadcast packet from their immediate parent, the T-Sink node based on mutual receiver-receiver synchronization [32,33]. After receiving the START message from the T-Mon Host, a T-Sink node broadcasts a time-synch command (Sync_Req, 1-hop broadcast packet) to every T-Sensor node in each lane.

The Sync_Req packet includes the date and time (*i.e.*, year, month, day, hour, minute, and second) of the T-Mon Host and the local time tick value (*u32Tick*) of the T-Sink node which is broadcasting this Sync_Req packet. Whenever a T-Sensor node receives the broadcast message, it takes a time stamp for the packet with its local TAR value and 32-bit local time tick value (*u32Tick*). Depending on the local processing state of each T-Sensor node, the processing latency for the received Sync_Req packet varies. Although the processing latency of each T-Sensor node in the same lane is diverse, as long as the processing latency is measured accurately, the *u32Tick* is updated and TAR and TACCR0 can be aligned correctly to the reference point.

Let us briefly describe the hardware design of each kind of node. All kinds of nodes in the TSN are based on a MSP430 Micro-Controller Unit (MCU) and CC2420 IEEE 802.15.4 compliant transceiver and CC2590 RF front-end amplifier from Texas Instruments [34]. To detect vehicles the T-Sensor node is equipped with a HMC1041Z magneto-resistive (MR) sensor from Honeywell [35]. The reasons why we adopt such an MR sensor are as follows: firstly, an MR sensor has been used in the vehicle detection field and proven with inductance loops for many years [17,20,21,29]. Unlike laser or PIR sensors, a MR sensor measuring perturbation of the Earth's ambient magnetic field (−6∼+6 gauss for HMC1041Z) by a vehicle doesn't detect other objects such as humans, and it is not easily interfered by weather as explained in Section 2. Although a small amount of day to day change in the Earth's magnetic field and the temperature change can be sources of false detections [36], they can be overcome by a simple algorithm, baseline adaptation which is explained in Section 3.3. Secondly, since it's small-sized (1 mm × 1 mm × 4 mm for HMC1041Z) and can be operated in low voltage level (2∼20 V for HMC1041Z) and low average current with the help of duty cycling, it makes possible to design a slim and small sized portable sensor node. To provide fault-tolerance to the failure of the wireless network after the start of the evaluation, the T-Sensor node has a back-up SD card on which to save undelivered packets. The T-Sink node is expendable for up to four sets of modules, each of which is responsible for a lane and consists of two communication modules connected via a Universal Asynchronous Receiver and Transmitter (UART). One communication module is for the upper tier network and the one is for the lower tier network. The T-BS-com is a gateway between the TSN and the T-Mon Host, and is attached with a back-up SD card in case the T-Mon Host fails.

**Figure 6.**
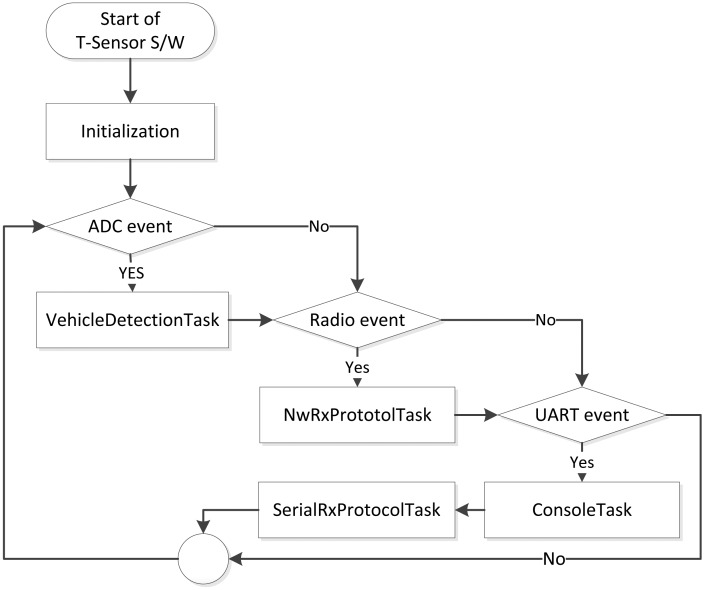
Overall operation procedure of T-Sensor node S/W.

Before ending this sub-section, the overall operation of a T-Sensor node is described. In our pervious project, S3 [29], through downscaling measurement, we have already demonstrated the battery-life time is over two years against the maximum traffic (155,000 vehicles/day or 1.8 vehicles/second) with careful hardware and software design. In this pilot project, we leave the T-sensor node always on, and the overall operation procedure of a T-Sensor node is described in Figure 6. When a T-Sensor node turns on, it prepares its operation in ‘*Initialization*’ by initializing several variables, the timer expiration period, the baseline (details in Section 3.3), and so on. The main routine consists of four tasks triggered by three event bits which are set in different interrupt service routines (ISRs). ADC event is set in an ADC ISR, which is executed by ADC conversion complete interrupt. Actually, a timer is set to expire every sampling period Ps in ‘Initialization’ phase, and the timer ISR starts ADC to sample the ambient magnetic field. Once ADC event is set, ‘*VehicleDetectionTask*’ is executed to decide two major detection events (APPROACH or DEPARTURE), and the details are explained in Section 3.3. The other two events, Radio and UART events are set by CC2420 and an internal UART controller, respectively. ‘*NwRxProtocolTask*’ is the main task for the network and application merged layer to process every received packet from the MAC layer. Depending on the destination address of the received packet, it takes the received packet and processes it in the application layer or just forwards the packet to a proper neighbor. If the destination of the packet is the current node and requires the response, it prepares the response packet and sends it to the requesting node. For the UART event, there are two tasks (*ConsoleTask*, *SerialRxProtocolTask*) to process the received character. The *ConsoleTask* processes each received character from any serial terminal emulator, and the *SeiralRxProtocolTask* is responsible for processing a serial packet generated from a local monitoring S/W which is a Graphic User Interface (GUI) software to monitor each node's internal state or to display the Earth magnetic field being measured by the node in real-time.

### 3.3. Vehicle Detection and Traffic Event Gathering

To measure traffic information such as speed, occupancy, and volume, the fundamental function is to accurately detect the approach or departure of a vehicle regardless of the kind of vehicle or the state it is in. A T-Sensor node is responsible for detecting an APPROACH or DEPARTURE event caused by a vehicle, and the main role of the T-Sink node is to take a time stamp for each event packet and to filter out unpaired event packets and retransmitted application-level packets. In this sub-section, the vehicle detection algorithm is summarized.

**Figure 7.**
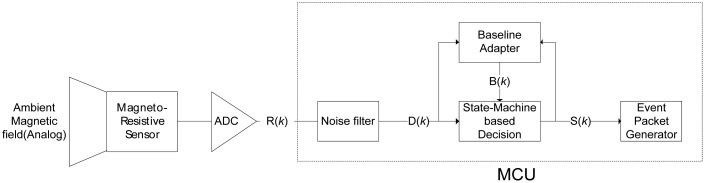
Overall flow of event detection in a T-Sensor node.

**Figure 8.**
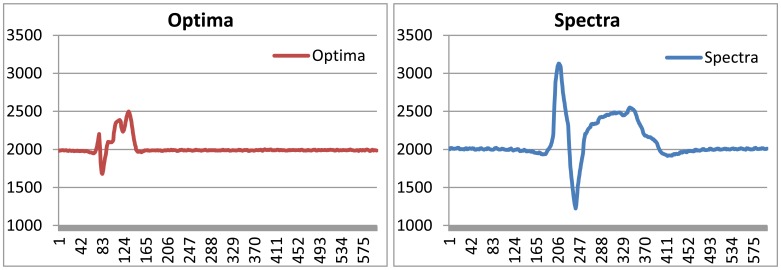
Refined data, D(k), gathered by prototype hardware.

To detect a vehicle, a magneto-resistive sensor is used and the overall detection flow is described in Figure 7. Since the Earth itself is a big magnet, there is an ambient magnetic field everywhere on Earth. When a T-Sensor node is turned on, it initializes the baseline for the ambient magnetic field. A ferrous object that drives by, such as a vehicle, distorts the ambient magnetic field. To monitor the distortion, the T-Sensor node samples the ambient magnetic field with a build-in Analog to Digital Converter (ADC) for a sampling period of Ps, which is decided by the maximum detectable speed and the minimum detectable length of a vehicle. The raw sampled data, R(*k*), usually has noise, which is filtered out by the noise filter. The refined data, D(*k*), is provided to both the Baseline Adapter block and the State Machine-based Decision block. It is worthwhile to refer to the gathered data (Figure 8), D(k), for different vehicles with different speeds from a prototype sensor node to continue to describe the remaining blocks. The State Machine-based Decision block decides the next state, S(*k*), based on D(*k*), B(*k*), and the current state. To provide hysteresis, two different thresholds are applied to the difference of D(k) and B(k) depending on the current state. The higher threshold is used to detect an approaching vehicle to a T-sensor node while the lower threshold is compared to detect a departing vehicle from the sensor node with the difference. To separate two consecutive vehicles is not so difficult, for there is enough space between two moving vehicles. Of course, the lower threshold should not be too small. If so, the sensor node regards them as a single vehicle. On the other hand, the Earth's magnetic field varies by small amounts day-to-day [36]. In addition, temperature changes on the sensor due to the movement of the sun and clouds result in the slow change of the sensed magnetic field [36]. To cope with those two sources of drift, the baseline adapter block is designed to adapt the baseline to those environmental changes. Depending on the output state of the State machine-based decision block, the baseline adapter modifies the baseline quickly or slowly to the recent trend of D(k). Once the next state, S(k), is decided by the decision block, the Event Packet Generator block prepares an event packet (APPROACH or DEPARTURE) with the current battery-level and sends the event to the T-Sink node.

When the T-Sink node receives the event packet, it updates the packet's timestamp field with its local time (tick) and forwards it to the T-BS-com. The T-BS-com gathers every packet from TSN and forwards them to the T-Mon Host via USB interface. For the case of failure of T-Mon Host, it saves only event packets in its local SD card.

The speed of a vehicle is easily calculated with v = (the separation of T-Sensor nodes in the same lane)/(time difference of APPROACH packets from them). The length of a vehicle is also estimated by v × (time interval between the paired APPROACH/DEPARTURE packets of one of T-Sensor nodes). Volume (or count) of traffic is measured by the paired APPROACH/DEPARTURE packets of one of the T-Sensor nodes. The occupancy is calculated by (time interval between APPROACH and DEPARTURE packets)/(unit time).

### 3.4. T-Mon Host

The T-Mon Host manages the overall evaluation procedure and all nodes in the TSN. Before starting the evaluation, it initiates the whole TSN by sending a START_EVALUATION packet with the reference time information to every T-Sink node via the T-BS-com node, which is connected to the T-Mon Host via USB interface. Once each T-Sink node receives the Start_Gathering packet from the T-Mon Host, it updates its local time to the received reference time and initiates a local time synchronization procedure in each lane by broadcasting a Sync_Req packet, as explained in Section 3.2. After initiating TSN, the T-Mon Host starts the evaluation. Then it not only gathers every packet from TSN, but also records a video of passing vehicles. Whenever the T-Mon Host receives an event packet, it calculates real-time traffic information such as speed, occupancy, and estimated length of the vehicle. In addition, it updates management information including the battery level of the sensor node that sent the event packet and the communication link state. Moreover, it relates the event information to the frame number in the video file. By mapping each event packet to a frame number, an operator can jump to the position in the video related to the event packet after finishing gathering traffic information for the evaluation. With this traceability, an operator is able to verify any abnormal traffic information.

Once finished gathering traffic information for the evaluation, the T-Mon Host provides post-processing to compare the traffic information measured by PES with that by VDS under evaluation. The T-Mon Host generates traffic information from the gathered event file in a Microsoft Excel file format. An operator can trace the position in the recorded video file for any event, since the T-Mon Host maps each event packet to the relevant frame number in the video file.

## 4. Implementation and Evaluation

We present the results of the implementation and evaluation of PES in this section.

### 4.1. Implementation and Deployments

Since the existing commercial sensor nodes could not meet the requirements (Table 1) described in Section 1, we implemented and manufactured each type of node, from enclosure and hardware platform to software, following the system architecture and design as in the previous section. The deployments for the evaluation consist of two phases. For the basic functional evaluation such as time synchronization, and reliability, the indoor deployment was done. As the second phase, to evaluate the accuracy of PES, it was deployed in the official ITS performance evaluation center in Korea which was located in a four-lane road and equipped with authorized facilities like high accurate VDSs, video recorder and tracker, and so on. To record different directions at the same time, two sets of PES were deployed. Each set of PES included four T-Sensor nodes for two for each lane, and two T-Sensor nodes in a lane were separated with 3 m to align with the distance of two reference laser detectors in the same lane (Figure 12). The detailed evaluation results for the two phased deployments are analyzed in the following sub-sections.

### 4.2. Functional Evaluation

#### 4.2.1. Time Synchronization

The overall time synchronization procedure is described in Figure 4 and Section 3.2. At first, we just considered synchronizing the date, time, and the software time tick, but the variation in the initial synchronization error was between 0 and 300 μs, which was quite large. We found that it was due to the ignorance of the alignment of the TAR and TACCR0 registers. Then we improved the time synchronization algorithm to take into account the alignment of those registers as well as the software time tick as explained in Figure 5. Finally, we reduced the variation of the initial time synchronization error to under several tens of microseconds.

The evaluation procedure consists of two phases: a software time tick and interrupt timing. Firstly, to make sure that the software time tick value is synchronized, T-Sensor nodes are asked to send a Ping_Res packet with their current software time tick values for a Ping_Req broadcast packet from the T-Sink node. We verified that T-Sensor nodes were synchronized using the aforementioned method, for they replied with the same tick values to the T-Sink node for the Ping_Req packet. Secondly, to evaluate the designed receiver-to-receiver time synchronization scheme in the timing offset level of a single timer interrupt, we needed a little more complex method using an oscilloscope. The local software time tick increased by 1 every 320 μs in the ISR for the Timer_A interrupt as described in Section 3.2. For every 10 cycles of the timer interrupt, we cleared a General Purpose Input Output (GPIO) pin for the first nine cycles and set the GPIO pin for the remaining 1 cycle in the ISR.

**Figure 9.**
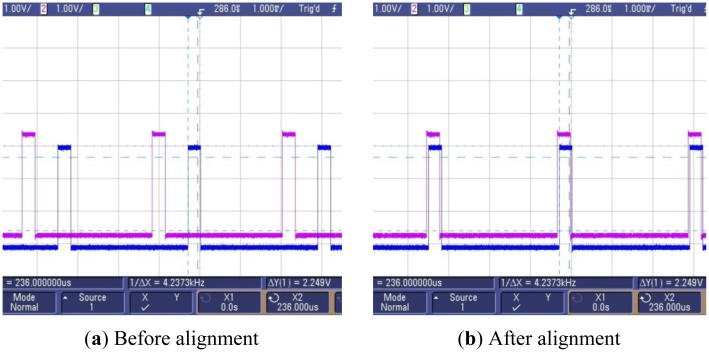
TAR, TACCR0 register alignment for synchronized periodic timer interrupt in two different T-Sensor nodes.

**Figure 10.**
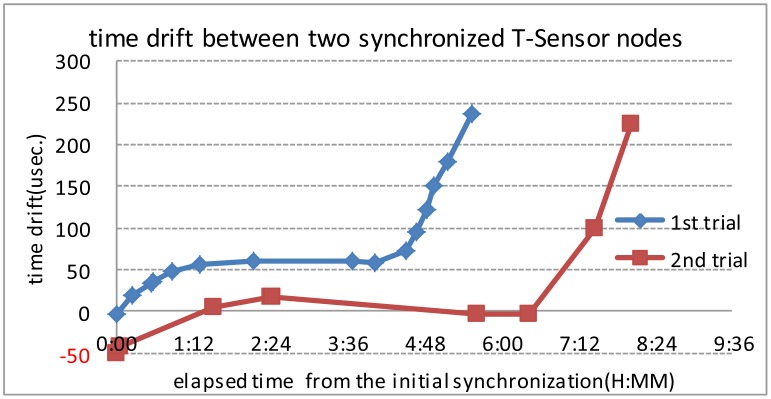
TAR, TACCR0 register alignment for synchronized periodic timer interrupt in 2 T-Sensor nodes.

The GPIO pins from two T-Sensor nodes are probed by an oscilloscope (Agilent 5000A) as in Figure 8. Ignoring the software time tick value, Figure 9(a) shows the misalignment of registers relevant to timers of two T-Sensor nodes. Through the time synchronization explained in Section 3.2, the offset between two T-Sensor nodes are aligned as Figure 9(b). The measurement result for the drift of the offset between two T-Sensor nodes after the initial synchronization is presented in Figure 10. For the target operation time of 6 hours of PES, the drift is under 300 μs.

#### 4.2.2. Reliability and Energy Consumption

**Figure 11.**
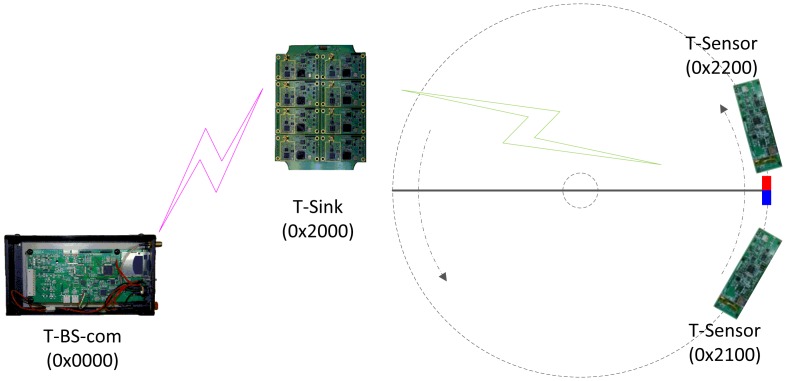
Indoor testbed for evaluating reliability.

To verify the reliability, we made a testbed to emulate a passing vehicle with a rotating arm to which a magnet is attached (Figure 11). Since we separate two T-Sensor nodes under the rotating magnet, they detect the magnet consecutively and report the events to T-BS-com node via T-Sink node.

We operated the testbed to emulate a passing vehicle every 715∼720 ms for 1∼24 hours several times and evaluated the functional reliability including detection and communication. T-Sensor nodes were configured to retransmit up to three times at the application layer based on MAC-level ACK, and the other nodes were set up to retransmit up to three times at the MAC-level. To measure the timing information, we used an oscilloscope (GDS-3254 [37]) and a TI's packet sniffer based on CC2420 and CC2520 [38].

As we described in Section 3, the T-BS-com node was designed to record every event (APPROACH or DEPARTURE) packet in its local SD card to support fault-tolerance, and we analyzed the recorded data. The event detection probability was measured to 100%, and the end-to-end packet delivery ratio for the event packets was measured to be between 99.1∼99.9% as shown in Table 4. Throughput for the end-to-end successful packet (29 bytes of MSDU) delivery was calculated from 638∼644 bps. We also measured the throughput between T-Sensor node and T-BS-com node for the real-time magnetic field monitoring packets (111 bytes of MSDU) which were gathered every 15.89 ms and reported from the T-Sensor node and it was measured to be 55.87 kbps. It is noticeable that it is not the maximum achievable throughput of the designed network stack. In addition, the application message (especially, EVENT message) delay from the detection to T-Sink node was measured to be 2.28 ms, 5.16 ms, 8.04 ms, and 10.92 ms for 1st time success, 1st retry success, 2nd retry success, and the last retry success based on MAC-level ACK and application-level retransmissions, respectively. Of course, since the upper tier network was based on MAC-level random back-off and retransmission, the delay from the T-sink to T-BS-com was variable between 5.07 ms and 7.40 ms. Therefore the overall latency of the EVENT message without any retransmission was measured to be from 2.28 + 5.07 = 7.35 ms to 2.28 + 7.40 = 9.68 ms. The long and variant latency of the upper tier network is due to the factors such as the random-backoff in LW-MAC and the transmission delay in UART connection in T-Sink node as described in Figure 2. However, the delays and jitters in the upper tier network do not affect the performance of the overall application.

As we described in Section 3, unlike other WSN applications, this application is not so strictly constrained by the battery-life time since the basic operation time of PES from the deployment is just six hours, and we leave the system always on. We evaluated the battery life time of PES with the same battery life time model in [29]. MCU is operated at 16 MHz with 10 mA, and the RF transceiver and CC2590 RF front end consume up to 22.8 (rx)∼39.5 mA(tx), and the magnetic sensor and relevant gain block including an amplifier draw the current up to 4.6 mA, and the worst-case current consumption of a T-Sensor node is 55.5 mA. We adopted 4,000 mAh Li-Ion rechargeable battery, and it's expected to operate about 74 h. Assuming six hours operation per evaluation, PES can be used for 12 evaluation periods without recharging.

### 4.3. Traffic Information

**Figure 12.**
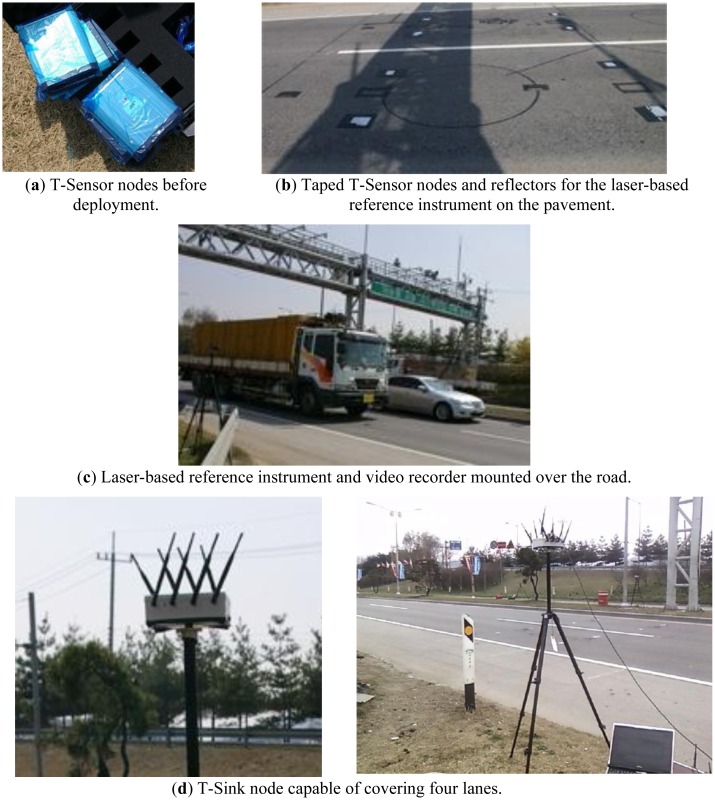

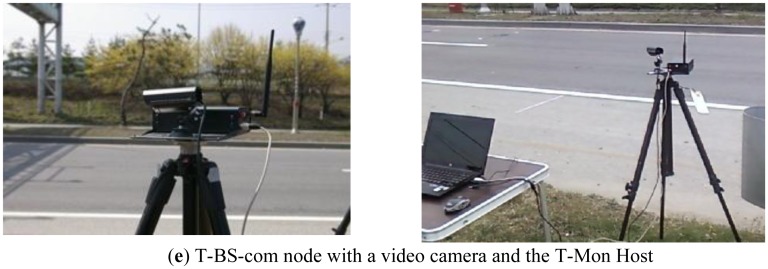
PES under evaluation in the KICT ITS performance evaluation center.

To evaluate the performance of PES, we used the ITS performance evaluation center of the Korea Institute of Construction Technology (KICT), which is located in Young-in, Gyeong-gi, Korea. The evaluation center is equipped with video recorders, a laser-based traffic monitor, loop detectors, and other equipment, and it can monitor traffic in two lanes each direction at the same time. Two sets of PES were deployed, evaluated in the center several times (Figure 12), and improved based on the results. In this sub-section, the final evaluation done on 18 April 2010 is analyzed in detail.

The following table (Table 5) shows evaluation durations and traffic volumes used to evaluate PES. For 40–70 min, 940–1,455 vehicles passed through the four lanes. As recommended by national regulations and considering PES as a reference (or evaluation) instrument [20], every one-minute period traffic information measured by PES is compared to that of the reference instrument (laser detector) in the evaluation center in Table 5. The average speed was found to be biased at about 1 km/h, so we compensated the amount of the biased speed for every minute of speed. Each piece of traffic information such as traffic volume, speed, and occupancy was measured for the evaluation duration by the reference instrument and PES, and was averaged over every minute. Therefore, average traffic information for every minute measured by PES was compared to that of the laser detector. Table 6 shows the Mean Absolute Percentage Error (MAPE) for every minute of traffic information for each lane, and the average MAPE for all of the lanes. Traffic volume accuracy, speed accuracy, and occupancy accuracy of PES were measured to be 1.03%, 1.61% and 6.01% respectively. Since PES was designed to be a portable system, we evaluated it in other provinces at different times, and it still showed similar performance.

As a reference, the performance of PES was compared indirectly with that of a commercial wireless vehicle detection system of Sensys Networks Inc. [39], which is a commercial WSN-based VDS. The accuracy assessment of a Sensys Wireless VDS is cited in [39] and some of the data is reproduced for comparison with PES. Since the metric used to evaluate the Sensys VDS is based on average volume accuracy and average speed accuracy over different 15-minute periods instead of 1 min periods, the measured data for PES is rearranged to follow the same metric. The average volume accuracy is compared first in Table 7. The MAPE for the volume accuracy of PES is between 0.7 % and 1.0 % for all four lanes, while that of Sensys VDS is 1.7%. In addition, the variation of the absolute error for PES is also smaller than that for the other. The speed accuracy of both systems is compared in Table 8. The MAPE for the speed accuracy of PES is between 0.3% and 1.3% for all four lanes, while that of Sensys VDS is 1.4%. The variation of the absolute speed error of PES is overall smaller than that of Sensys VDS.

When the PES is compared indirectly with the Sensys VDS from the traffic volume and speed accuracy viewpoints, PES shows better performance. If we consider measurement (volume count or speed) accuracy for each QN (15-Minute Quarter Number) with volume count (an index of traffic density) in the Ground Truth (GT) column in Table 7, it is difficult because the performances of both systems are highly dependent on traffic density. However, for a fairer comparison, Sensys VDS and PES need to be deployed in the same site in order to measure the traffic information of the same vehicles and evaluated by the same reference instrument.

## 5. Conclusions

Since maintaining the accuracy of VDS is crucial to support smart transportation systems, a governmental office regularly manages the deployed VDS. However, the conventional VDS evaluation systems based on video-frame analysis, piezoelectric cables, or lasers have the drawbacks of high installation and maintenance costs, long analysis time, lack of scalability (*i.e.*, difficulty to detect multiple lanes), and lack of objectivity of evaluation. To alleviate the aforementioned drawbacks of the conventional systems, this paper proposed an overall system architecture for a Portable Vehicle Detector Evaluation System (PES) based on wireless sensor networks. The paper describes the customized two-tiered network architecture including Light Weight MAC (LW-MAC), Level-based Static Routing (LSR), and time synchronization scheme, as well as the vehicle detection algorithm. Based on a field implementation of PES, it was demonstrated by extensive evaluations to produce accurate traffic information such as volume count, speed, and occupancy. Especially, PES was evaluated to measure volume count and speed at the accuracy of 99% and 98%, respectively. Future work includes the improvement of accuracy of the measured traffic information as well as the improved time synchronization protocol with piggy-backing the time synchronization information to the detection packet.

## Figures and Tables

**Table 1.** Requirements for a new portable evaluation system.

**Requirements**	**Descriptions**
Cost	Cheaper than the existing instruments
Set-up time	Easy and fast to set up the system in the field to minimize traffic interference
Accuracy	To evaluate the pre-installed VDSs, the accuracy should be very high
Objectivity	By minimizing human interventions during the evaluation procedure, evaluation objectivity should be assured
Scalability	The system should evaluate multiple lanes at the same time
Traceability	To assist the video analysis of the information gathered for each vehicle, the recorded video should be tracable by a time stamp.
Real-time monitoring	During the evaluation procedure, instantaneous evaluation results such as volume, occupancy, and speed can be shown in real-time
Fault tolerance	Despite partial faults of the system, the evaluation in progress should be completed. Even after wireless interferences, the traffic information can be recovered by post-processing.

**Table 2.** Overall comparison between the existing portable evaluation systems (or VDSs) and PES.

	**Video Frame**	**Portable Laser**	**Piezoelectric Cable**	**Existing MR VDSs**	**PES**
Cost	High	High	High (Maintenance)	Economic	Economic
Easy/fast to install	Yes	Yes	Yes	Yes	Yes
Accuracy	Good	High	High	High	High
Objectivity	Low	High	High	High	High
Multi-lane Scalability	Yes	Difficult	Difficult	Yes	Yes
Traceability	Yes	Yes	Yes	No	Yes
Real-time monitoring	Yes	Yes	Yes	No	Yes
Fault-tolerance	Yes	Yes	Yes	Some	Yes

**Table 3.** Notation used in Figures 4 and 5 for the T-Sensor node time synchronization.

**Notation**	**Description**
T_R_	Broadcast (Sync. Req.) message receiving time, Reference point
T_S_	Synchronized time to the reference point
K	the received software time tick from T-Sink Node. Appended in Sync_Req packet
SN	S1, S2, S3: T-Sensor node's local software time tick before the synchronization
TAR	Timer_A register
TACCR0	Timer_A Capture/Compare 0 Register. Timer interrupt is occurred whenever TAR is equal to TACCR0
Lp	Processing Latency, the time interval from T_B_ to the beginning of the packet processing
Ls	Processing Latency measured by the local software timer (incremented in ISR(interrupt service routine) triggered by TAR and TACCR0)
Ds	Duration of a software time tick

**Table 4.** End-to-End packet delivery ratio.

**Evaluated Node**	**Evaluation Time (hh:mm:ss)**	**PDR (%)**	**Throughput (bps)**
0x2100	22:03:02-22:06:18	99.2634	638.00
0x2200		99.2634	638.00
0x2100	02:47:56∼09:29:12	99.4214	644.05
0x2200		99.4630	644.32
0x2100	10:13:00∼10:55:39	99.1822	642.96
0x2200		99.0541	642.16
0x2100	11:34:42∼14:17:49	99.9926	638.16
0x2200		99.9926	638.16

**Table 5.** Evaluation durations and traffic volume.

**Lanes**	**Evaluation Time**	**Traffic Volume**
Northbound lane #1 (N1)	13:18–14:59	1,273
Northbound lane #2 (N2)	13:17–15:10	1,392
Southbound lane #1 (S1)	13:07–15:16	1,455
Southbound lane #2 (S2)	13:07–15:16	940

**Table 6.** Evaluation results.

**Lanes**	**Mean Absolute Percentage Error for Every Minute of Traffic Information**			

	**Traffic volume**	**Average Speed**	**Compensated Average Speed**	**Occupancy**

N1	1.3	2.28	1.71	6.11
N2	1.01	1.76	1.65	6.05
S1	0.86	1.02	1.13	5.28
S2	0.95	3.04	1.96	6.60

**Average**	**1.03**	**2.02**	**1.61**	**6.01**

**Table 7.** Volume count accuracy comparison between PES and Sensys VDS [39].

**11-19 September 2006**				**PES, 18 April 2010**							

**Sensys VDS[39]**				**N1**				**S1**			

QN^1)^	Count		Abs.Error(%)	QN^1)^	Count		Abs.Error(%)	QN^1)^	Count		Abs.Error(%)
		
	GT^2)^	VDS			GT^3)^	PES			GT^3)^	PES	

1	218	209	4.1	P1	187	187	0.0	P1	149	148	0.7
2	228	234	2.6	P2	170	169	0.6	P2	167	167	0.0
3	213	217	1.9	P3	175	173	1.1	P3	184	181	1.6
4	248	242	2.4	P4	189	188	0.5	P4	170	171	0.6
5	212	207	2.4	P5	199	196	1.5	P5	165	165	0.0
6	193	198	2.6	P6	193	192	0.5	P6	171	171	0.0
7	204	203	0.5	P7	160	159	0.6	P7	153	156	2.0
8	233	229	1.7					P8	174	173	0.6
9	236	238	0.8					P9	122	121	0.8
				
10	216	210	2.8	**MAPE**			**0.7**	**MAPE**			**0.7**
				
11	200	194	3.0	**Var. of Abs. Error**			**0.2**	**Var. of Abs. Error**			**0.5**
				
12	216	212	1.9	**N2**				**S2**			
				
13	227	225	0.9	QN^1)^	Count		Abs.Error(%)	QN^1)^	Count		Abs.Error(%)
					
14	205	206	0.5		GT^3)^	PES			GT^3)^	PES	
				
15	184	177	3.8	P1	188	185	1.6	P1	111	110	0.9
16	219	218	0.5	P2	174	176	1.1	P2	100	101	1.0
17	275	271	1.5	P3	168	170	1.2	P3	112	113	0.9
18	199	200	0.5	P4	169	170	0.6	P4	117	117	0.0
19	183	178	2.7	P5	198	198	0.0	P5	124	126	1.6
20	179	177	1.1	P6	183	184	0.5	P6	112	111	0.9
21	239	237	0.8	P7	200	199	0.5	P7	98	99	1.0
22	197	197	0.0	P8	112	113	0.9	P8	94	95	1.1
23	204	204	0.0					P9	72	71	1.4

**MAPE**			**1.7**	**MAPE**			**0.8**	**MAPE**			**1.0**

**Var. of Abs. Error**			**1.4**	**Var. of Abs. Error**			**0.3**	**Var. of Abs. Error**			**0.2**

Notes:

QN^1)^: 15-Minute Quarter Number

GT^2)^: Video Ground Truth

GT^3)^: Video/Laser Ground Truth

**Table 8.** Speed accuracy comparison between PES and Sensys VDS [39].

**11-19 September 2006**				**PES, 18 April 2010**							

**Sensys VDS [39]**				**N1**				**S1**			

QN^1)^	Avg. Speed (mph)		Abs.Error(%)	QN^1)^	Avg. Speed (mph)		Abs.Error(%)	QN^1)^	Avg. Speed(mph)		Abs.Error(%)
		
	GT^2)^	VDS			GT^3)^	PES			GT^3)^	PES	

2	67.7	67.4	0.4	P1	37.3	37.3	0.0	P1	43.3	43.4	0.2
5	66.7	64.7	3.0	P2	41.9	42.0	0.2	P2	40.6	40.6	0.0
10	64.4	64.4	0.0	P3	42.2	42.0	0.5	P3	42.1	42.7	1.4
13	66.6	65.9	1.1	P4	42.2	42.2	0.0	P4	42.1	42.4	0.7
17	67.6	65.6	3.0	P5	41.3	41.5	0.5	P5	40.7	41.2	1.2
21	66.8	66.1	1.0	P6	41.5	41.3	0.4	P6	41.8	42.2	1.0
				P7	41.1	41.3	0.5	P7	41.8	42.1	0.7
								P8	43.4	43.8	0.9
								P9	43.1	43.2	0.2
				
				**MAPE**			**0.3**	**MAPE**			**0.7**
				
				**Var. of Abs. Error**			**0.1**	**Var. of Abs. Error**			**0.2**
				
				**N2**				**S2**			
				
				QN^1)^	Avg. Speed (mph)		Abs.Error(%)	QN^1)^	Avg. Speed (mph)		Abs.Error(%)
					
					GT^3)^	PES			GT^3)^	PES	
				
				P1	34.1	34.5	1.2	P1	40.9	40.4	1.2
				P2	37.2	37.5	0.8	P2	39.8	39.6	0.5
				P3	41.3	41.3	0.0	P3	41.4	40.7	1.7
				P4	39.6	39.7	0.3	P4	40.7	39.9	2.0
				P5	38.0	38.0	0.0	P5	41.4	40.7	1.7
				P6	37.5	38.0	1.3	P6	39.8	39.4	1.0
				P7	37.8	39.2	3.7	P7	40.7	40.5	0.5
				P8	37.2	38.1	2.4	P8	43.3	42.9	0.9
								P9	41.6	40.8	1.9

**MAPE**			**1.4**	**MAPE**			**1.2**	**MAPE**			**1.3**

**Var. of Abs. Error**			**1.7**	**Var. of Abs. Error**			**1.6**	**Var. of Abs. Error**			**0.3**

Notes:

QN^1)^: 15-Minute Quarter Number

GT^2)^: Laser Gun Ground Truth

GT^3)^: Laser Ground Truth
